# Sildenafil reduces neuroinflammation and restores spatial learning in rats with hepatic encephalopathy: underlying mechanisms

**DOI:** 10.1186/s12974-015-0420-7

**Published:** 2015-10-29

**Authors:** Vicente Hernandez-Rabaza, Ana Agusti, Andrea Cabrera-Pastor, Santos Fustero, Oscar Delgado, Lucas Taoro-Gonzalez, Carmina Montoliu, Marta Llansola, Vicente Felipo

**Affiliations:** Laboratory of Neurobiology, Centro de Investigación Príncipe Felipe, Calle Eduardo Primo Yufera, 3, 46012 Valencia, Spain; Fundación Investigación Hospital Clínico de Valencia. Instituto de Investigación Sanitaria-INCLIVA, Valencia, Spain; Laboratorio de Moleculas Orgánicas, Centro de Investigación Príncipe Felipe, Valencia, Spain; Departamento de Química Organica, Universidad de Valencia, Valencia, Spain

**Keywords:** cGMP, Neuroinflammation, Hepatic encephalopathy, Cognitive impairment, Sildenafil treatment

## Abstract

**Background:**

There are no specific treatments for the neurological alterations of cirrhotic patients with minimal hepatic encephalopathy (MHE). Rats with MHE due to portacaval shunt (PCS) show impaired spatial learning. The underlying mechanisms remain unknown. The aims of this work were to assess: (a) whether PCS rats show neuroinflammation in hippocampus, (b) whether treatment with sildenafil reduces neuroinflammation and restores spatial learning in PCS rats, and (c) analyze the underlying mechanisms.

**Methods:**

Neuroinflammation was assessed by determining inflammatory markers by Western blot. Phosphorylation of MAP-kinase p38 was assessed by immunohistochemistry. Membrane expression of GABA and glutamate receptors was analyzed using BS3 cross-linker. Spatial learning was analyzed using the radial and Morris water mazes. To assess if sildenafil reverses the alterations, rats were treated with sildenafil in the drinking water.

**Results:**

PCS rats show increased IL-1β and TNF-α levels and phosphorylation (activity) of p38 in hippocampus. Membrane expression of subunits α1 of GABA_A_ receptor and GluR2 of AMPA receptor are increased in PCS rats, while subunits GluR1 of AMPA receptors and NR1 and NR2a of NMDA receptors are reduced. PCS rats show reduced spatial learning in the radial and Morris water mazes. Sildenafil treatment normalizes IL-1β and TNF-α levels, p38 phosphorylation, and membrane expression of GABA_A_, AMPA, and NMDA receptors and restores spatial learning.

**Conclusions:**

Increased IL-1β alters GABAergic and glutamatergic neurotransmission in hippocampus and impairs spatial learning in rats with MHE. Sildenafil reduces neuroinflammation and restores learning. Phosphodiesterase-5 inhibitors may be useful to improve cognitive function in patients with MHE.

## Background

Minimal hepatic encephalopathy (MHE) is not clinically evident but can be unveiled using psychometric tests. MHE is suffered by around 40 % of cirrhotic patients (several million people). Patients with MHE show psychomotor slowing, attention deficits, mild cognitive impairment, and visuo-spatial incoordination [1]. These deficits affect their quality of life, progresses to clinical HE, and reduces life span.

Hyperammonemia and inflammation act synergistically to induce mild cognitive and motor impairment in MHE [2–4]. Chronic hyperammonemia per se induces neuroinflammation that contributes to the neurological alterations in MHE [5]. The mechanisms responsible for some specific neurological alterations in MHE are beginning to be clarified in animal models [6–8].

Rats with portacaval shunts (PCS) are a good model of MHE that reproduces cognitive and motor alterations present in patients with MHE [9]. In PCS rats, neuroinflammation in cerebellum mediates the impairment in the ability to learn a Y-maze task [10] and in basal ganglia mediates hypokinesia [11]. Different types of cognitive functions and of learning or memory are modulated by different mechanisms involving different pathways and brain areas. While the ability to learn a Y-maze task is mainly modulated in cerebellum by the glutamate-NO-cGMP pathway, spatial learning, and memory are mainly modulated in hippocampus by different mechanisms. Therefore, restoring spatial learning and memory requires normalizing these mechanisms in hippocampus. Spatial learning in the Morris water maze is impaired in PCS rats [12] but the underlying mechanisms and the role of neuroinflammation have not been studied in detail.

We hypothesize that these mechanisms could be mediated by increased interleukin 1β (IL-1β) in hippocampus. Sustained expression of IL-1β in hippocampus impairs spatial learning and memory [13]. Wang et al. [14] showed that IL-1β, through activation of p38 mitogen-activated protein kinase, leads to enhanced membrane expression of GABA_A_ receptors and of an associated inhibitory current in hippocampus and impaired learning. Blocking GABA_A_ receptors prevented the impairment of learning induced by IL-1β.

The above studies suggest that impaired spatial learning in PCS rats could be due to neuroinflammation in hippocampus and that reducing it could restore spatial learning.

The phosphodiesterase-5 inhibitor sildenafil reduces neuroinflammation in hippocampus and improves cognitive performance in APP/PS1 transgenic mice model of Alzheimer’s disease [15]. Sildenafil also reduces neuroinflammation and IL-1β levels in cerebellum in an inflammatory model of demyelination in mice [16] and restores learning of a Y-maze conditional task, mainly modulated in cerebellum, in PCS rats [17]. It has not been assessed whether sildenafil reduces neuroinflammation and/or improves spatial learning in rats with MHE due to PCS.

The aims of this work were the following:

- assess whether PCS rats show neuroinflammation in hippocampus;
- assess whether treatment with sildenafil reduces hippocampal neuroinflammation and restores spatial learning in PCS rats, and
- analyze the underlying mechanisms, including changes in membrane expression of glutamate and GABA_A_ receptors.

## Methods

### Portacaval anastomosis and treatment with sildenafil

Male Wistar rats (220–240 g) subjected to end-to side portacaval anastomosis as described by Lee and Fisher [18]. Control rats were sham operated. Adequate measures were taken to minimize pain and discomfort to the animals. The experiments were approved by the Comite de Experimentación y Bienestar Animal (CEBA) of our center and performed in accordance with guidelines of the Directive of the European Commission (2010/63/EU) for care and management of experimental animals.

**Fig. 1:**
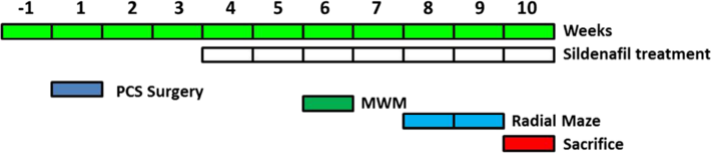
Scheme showing the experimental design

Rats were treated with sildenafil (50 mg/L, purified from Viagra pills) in the drinking water, beginning 4 weeks after PCS surgery until sacrificed. The experimental design is summarized in Fig. 1.

### Analysis of protein content in hippocampus by Western blot

Homogenates of hippocampus were subjected to immunoblotting as in [19]. Primary antibodies were against IL-4, IL-10 (1:2000) α1 and α5 subunits of GABA_A_ receptors (1:500) from Abcam (Cambridge, UK); IL-1β and TNF-α (1:500) from R&D SYSTEMS (Minneapolis, USA); GluR1(1:1000) from Calbiochem (Darmstadt, Germany); GluR2 (1:1000) from Chemicon (Temecula, CA, USA); NR1 (1:1000) from BD Biosciences (San Jose, CA, USA), and NR2A (1:1000) from Upstate Biotechnology (New York, NY, USA). As control for protein loading, the membranes were incubated with anti-actin (Abcam, Cambridge, MA; 1:1000). Secondary antibodies were anti-rabbit, anti-goat, or anti-mouse IgG (1:2000) conjugated with alkaline phosphatase (Sigma, St. Louis, MO). The images were captured using the ScanJet 5300C (Hewlett-Packard, Amsterdam, Netherlands) and band intensities quantified using the Alpha Imager 2200, version 3.1.2 (AlphaInnotech Corporation, San Francisco).

Analysis of surface expression of receptors by cross-linking with BS_3_ was performed as described in [20]. Hippocampi were dissected and transversal slices (400 μm) were obtained using a chopper. Slices were added to tubes containing ice-cold standard buffer with or without 2 mM BS_3_ (Pierce, Rockford, IL) and incubated for 30 min at 4 °C. Cross-linking was terminated by adding 100 mM glycine (10 min, 4 °C). The slices were homogenized by sonicating for 20 s. Samples treated or not with BS_3_ were analyzed by Western blot. The surface expression of each receptor was calculated as the difference between the intensity of the bands without BS3 (total protein) and with BS3 (non-membrane protein).

### Spatial learning

Spatial learning in the Morris water maze. The test was carried out as described in [12]. After pre-training, rats were trained to learn the fixed location of the hidden platform during 4 days (three swims per day). Rats were allowed a maximum of 120 s to find the platform. The time needed to find the platform was recorded as a measure of learning.

Spatial learning in the 8-arms radial maze was assessed as described in [21]. Training was performed during 10 days (three trials per day). The number of working memory errors (working errors, visits to arms already visited in the same trial) were recorded and are presented in blocks of 2 days.

#### Immunohistochemistry

Immunohistochemistry was performed as described in [5]. Five to six animals per group were anesthetized and transcardially perfused with 150 mL of saline, followed by 250 mL of 0.4 % paraformaldehyde in 0.1 M phosphate buffer pH 7.4. Brains were removed and immersed for 24 h at 4 °C in the same fixative. Free-floating sections (30 μm) were cut through the hippocampus. For the DAB sections, sections were incubated with biotinylated secondary antibodies and avidin-biotin-HRP complex (ABC kit, Vector, CA, USA) followed DAB-H_2_O_2_ substrate. For immunofluorescence, sections were incubated with the corresponding fluorescent secondary antibody followed by incubation with the nuclear marker DAPI. The stained sections were mounted on slides and coverslipped. Primary antibodies used were the same as for Western blot but with (1:200) dilutions for IL-1β, receptor of IL1β, p38-MAPK from Millipore (Darmstadt, Germany). The secondary biotinylated antibodies (1:200) from Vector Laboratories (Burlingame, CA, USA) and secondary fluorescent antibodies (1:500) from Invitrogen (Oregon, USA) were used.

### Analysis of microglial activation

Microglial activation was assessed in the CA1 region of hippocampus by immunohistochemistry as in [22]. Microglia was stained with anti-Iba 1 (1:200) as above and the total perimeter of individual microglial cells was measured using Image J software (ImageJ 1.48v, http://imagej.nih.gov/ij) by counting the number of pixels surrounding the cell. Eight sections per rat were analyzed in three rats per group and the mean perimeter of microglial cells was calculated.

Phosphorylation of MAP kinase p38 in hippocampus was assessed by immunohistochemistry as described above using an antibody against phosphorylated p38 (1:200) from Millipore (Darmstadt, Germany). This approach was chosen because sacrifice of rats and hippocampus homogenization alters p38 phosphorylation. Rats were perfused transcardially with 0.4 % paraformaldehyde that fixes the tissue and preserves p38 phosphorylation as present in vivo.

### Statistical analysis

The results are presented as mean ± standard error of mean (SEM). The data were analyzed by one-way ANOVA followed Dunnett’s *T* test. *p* values lower than 0.05 were considered statistically significant. Statistical analysis was performed using the Graph Pad Prism 4 software (GraphPad Software Inc. San Diego, CA).

## Results

### PCS rats show increased levels of IL-1β, TNF-α and phosphorylation of p38 in hippocampus, which are reversed by sildenafil

**Fig. 2:**
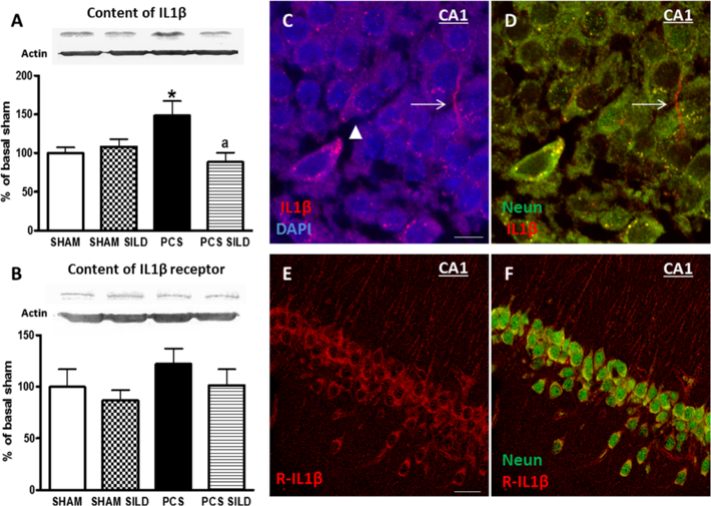
IL1β levels are increased in hippocampus of PCS rats and are normalized by treatment with sildenafil. IL1β (**a**) and IL1β receptor (**b**) amount in the hippocampus were determined by Western blot in sham and PCS treated with saline or sildenafil (SILD). Representative images of the Western blots are shown. Values are the mean ± SEM of 12 rats per group. **c**–**f** Confocal images showing the expression of IL-1β and IL-1 receptor in neurons of the CA1 region of hippocampus of PCS rats. **c**, **d** IL1β signal (*red*), nucleus marker DAPI (*blue*), and neuronal marker Neun (*green*). Note the expression of IL1β in the CA1 region both in neurons (*arrow heads*) and no neuronal cells (*arrows*). **e**, **f** IL1β receptor signal (*red*) and Neun (*green*). Note the expression of the IL1β receptor in the pyramidal CA1 neurons. Scale bar 10 μm (**c**, **d**) and 20 μm (**e**, **f**)

The content of IL-1β in hippocampus, as analyzed by Western blot (Fig. 2a), was increased in PCS compared to control rats (149 ± 19 %, *p* < 0.05). Treatment with sildenafil did not affect IL-1β in control rats (108 ± 10 %) but reduces it to normal levels in PCS rats (88 ± 12 %, *p* < 0.05).

A similar effect was observed for TNF-α and for IL-10, while IL-4 is not significantly affected in PCS rats or by sildenafil (Table 1).

**Table 1:** IL-1β, TNF-α, and IL-10 contents are increased in hippocampus of PCS rats and are normalized by treatment with sildenafil

Hippocampus	Sham	Sham + SILD	PCS	PCS + SILD
TNF-α	100 ± 17	125 ± 13	144 ± 9*	103 ± 7^aa^
	*n* = 9	*n* = 10	*n* = 9	*n* = 9
IL-1β	100 ± 7	108 ± 10	149 ± 19*	88 ± 12^a^
	*n* = 12	*n* = 13	*n* = 12	*n* = 12
IL-4	100 ± 9	109 ± 14	112 ± 12	93 ± 13
	*n* = 10	*n* = 10	*n* = 10	*n* = 11
IL-10	100 ± 11	106 ± 7	144 ± 13*	105 ± 10^a^
	*n* = 11	*n* = 11	*n* = 10	*n* = 10

Values are the mean ± SEM of the number of rats indicated (*n*). Values significantly different from sham rats are indicated by asterisks. Values significantly different from PCS rats not treated with sildenafil are indicated by *a*

*p < 0.05; ^a^ *p* < 0.05; ^aa^ *p* < 0.01

The content of the receptor for IL-1β is slightly but not significantly increased in PCS rats (122 ± 15 %) and was not affected by sildenafil in control or PCS rats (Fig. 2b).

Both IL-1β (Fig. 2c, d) and its receptor (Fig. 2e, f) are present in neurons of the CA1 region of hippocampus, as revealed by immunohistochemistry.

**Fig. 3:**
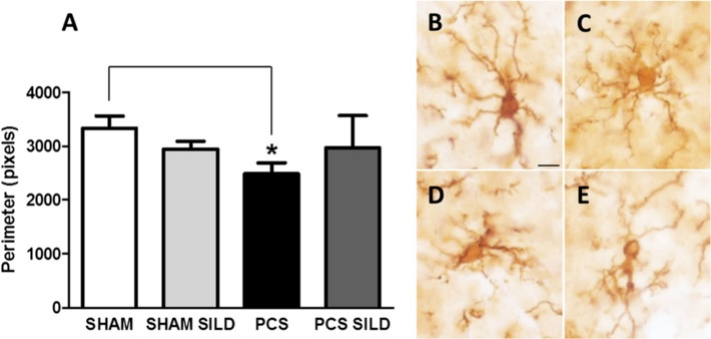
Microglia is activated in hippocampus of PCS rats. Sildenafil reduces microglial activation. **a**. Microglial activation was analyzed in the CA1 region of hippocampus by measuring the perimeter of microglial cells stained with Iba-1. Values are given as number of pixels surrounding microglial cells. Values are the mean ± SEM of eight sections per rats and three rats per group. Representative images of microglia morphology as stained with Iba-1 are shown for sham controls (**b**), sham control rats treated with sildenafil (**c)**, PCS rats (**d**) and PCS rats treated with sildenafil (**e**). Scale bar (**b**–**e**) =10 μm

To assess whether reduction of IL-1β levels by sildenafil in PCS rats is associated with reduced microglial activation, we analyzed it by immunohistochemistry. PCS rats show a mild activation of microglia in hippocampus which is reflected in a reduction of the perimeter which was 3336 ± 225 pixels in control rats and was reduced (*p* < 0.05) in PCS rats to 2481 ± 211. In PCS rats treated with sildenafil, the perimeter returned to normal values (2972 ± 596 pixels), indicating that it reduces microglial activation in PCS rats (Fig. 3).

**Fig. 4:**
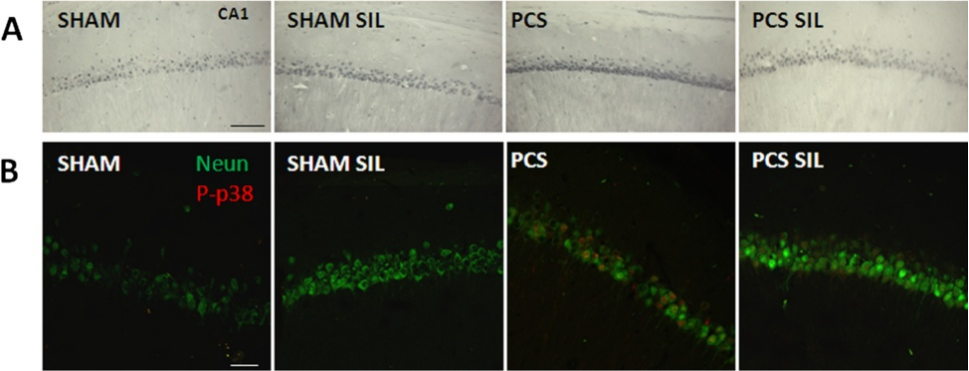
Phosphorylation of p38 is increased in hippocampus of PCS rats and is normalized by treatment with sildenafil. **a**, **b** Representative images of phosphorylated p38 (P-p38) in the CA1 region of the dorsal hippocampus in control and PCS rats, treated with vehicle or sildenafil (SIL). **a** Phosphorylated p38 immunostained with DAB. Note the higher intensity in PCS rats. **b** Confocal images of the immunostaining for Neun (*green*) and phosphorylated p38 (*red*) in neurons of the CA1 region. Scale bar 100 μm in (**a**) and 20 μm in (**b**)

Phosphorylation of p38, as analyzed by immunohistochemistry was increased in the CA1 region of hippocampus of PCS compared to control rats. Treatment with sildenafil strongly reduced the phosphorylation of p38 both in control and PCS rats (Fig. 4a).

Figure 4b shows by double fluorescence staining that phosphorylation of p38 is mainly located in neurons and is very low in neurons of CA1 region of hippocampus in control rats, is strongly increased in PCS rats and is reduced by treatment with sildenafil.

### Membrane expression of GABA_A_ and GluR2 receptors is increased and that of NR1, NR2A, and GluR1 are reduced in hippocampus of PCS rats. All these changes are reversed by sildenafil

Membrane expression of different GABA_A_ and glutamate receptor subunits in hippocampus was quantified using the BS3 method.

**Fig. 5:**
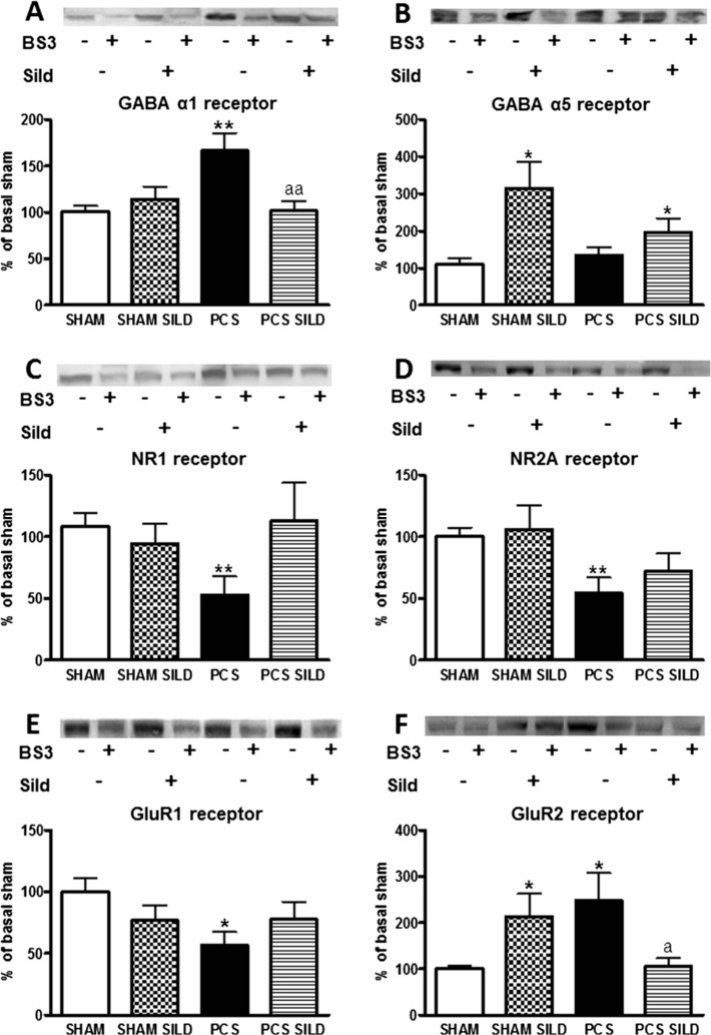
Surface receptor expression was assessed in SHAM and PCS treated or not with sildenafil (SILD). The membrane expression of the alpha 1 subunit of GABA_A_ receptor (**A-B**), NR1 and NR2a subunits of NMDA receptors (**C-D**) and GluR1 and GluR2 subunits of AMPA receptors (**E-F**) is altered in hippocampus of PCS rats and is normalized by treatment with sildenafil. Membrane expression of each subunit in hippocampus was analyzed using the BS3 crosslinker procedure as described in methods. Samples incubated in the absence or presence of BS3 were subjected to Western blotting using antibodies for each of the subunits. BS3 or sildenafil presence or absence was expressed as “+” or “-“, respectively. Representative images are shown. Samples in the absence of BS3 represent the total amount of each protein. Samples in the presence of BS3 represent the non-membrane fraction. The intensities of the bands were quantified and membrane expression was calculated as the difference of intensity between samples without and with BS3. Values are expressed as percentage of control rats and are the mean ± standard errors of 14–18 rats per group. Values significantly different from control rats are indicated by *asterisks* and from PCS rats by “*a*”. *p < 0.05;**p < 0.01; ^a^ *p* < 0.05

The expression in membrane of the alpha 1 subunit of GABA_A_ receptors was increased in PCS rats to 166 ± 19 % (*p* < 0.05) of controls while that of the alpha 5 subunit was not affected (Fig. 5a, b).

Concerning NMDA receptors, both the membrane expression of NR1 and NR2A subunits were reduced (*p* < 0.05) in PCS rats to 53 ± 15 and 54 ± 13 % of controls, respectively (Fig. 5c, d).

Concerning AMPA receptors, the membrane expression of the GluR1 subunit was reduced (*p* < 0.05) in PCS rats to 56 ± 1 % while the expression of the GluR2 subunit was strongly increased to 227 ± 60 % of controls (Fig. 5e, f).

Treatment with sildenafil normalized the membrane expression of all the subunits altered in PCS rats: alpha 1 of GABA_A_ receptors, NR1 and NR2A of NMDA receptors, and GluR1 and GluR2 of AMPA receptors (Fig. 5a–f).

**Fig. 6:**
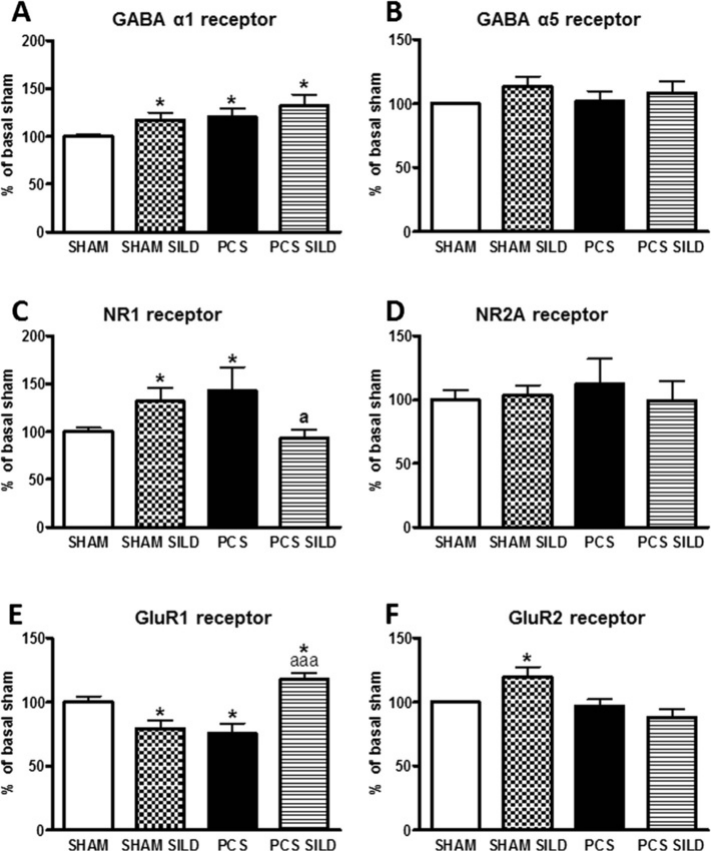
Total receptor expression was assessed in SHAM and PCS treated or not with sildenafil (SILD). Total amount of the alpha 1 subunit of GABAA receptor (**A-B**), NR1 and NR2a subunits of NMDA receptors (**C-D**) and GluR1 and GluR2 subunits of AMPA receptors (**E-F**) in hippocampus of PCS and SHAM rats treated or not with sildenafil. Experiments were the same shown in Fig. 5 but only the samples incubated in the absence of BS3 (total amount) are presented. The intensities of the bands were quantified and expressed as percentage of control rats. Values are the mean ± standard errors of 14–18 rats per group. Values significantly different from control rats are indicated by asterisks and from PCS rats by *a*. *p < 0.05; ^a^ *p* < 0.05; ^aaa^ *p* < 0.001

It is noteworthy that changes in the above subunits in PCS rats are specific for membrane expression, while the changes in total amount of each subunit in the whole hippocampus homogenate were different: alpha 1 and NR1 subunits are slightly increased, NR2A and GluR2 are not affected while GluR1 is reduced (Fig. 6).

### Spatial learning is impaired in PCS rats and is restored by sildenafil

**Fig. 7:**
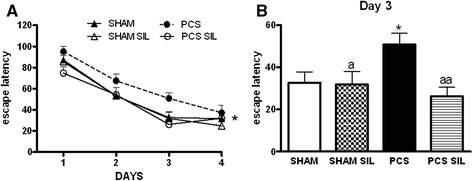
Spatial learning in the Morris water maze is impaired in PCS rats and is restored by treatment with sildenafil. Spatial learning ability in the Morris water maze was assessed in sham and PCS rats, treated with vehicle or sildenafil (SIL). **a** Escape latencies (in seconds) to reach the platform during the different sessions. **b** Escape latencies (in seconds) in the third session. Values are the mean ± SEM of 15–18 rats per group. Values significantly different from control rats are indicated by *asterisks* and from PCS rats by *a*. *p < 0.05; ^a^ *p* < 0.05; ^aa^ *p* < 0.01

PCS rats show reduced learning ability in the Morris water maze. As shown in Fig. 7a, all groups of rats learned to find the platform and the latency to reach it was reduced along the four training days. However, learning ability was significantly reduced in PCS rats (*p* < 0.05 versus controls). The latency to find the platform on day 3 is shown in Fig. 7b. Control rats needed 34 ± 5 s to find the platform, while PCS rats needed more time (51 ± 5 s, *p* < 0.01).

Treatment with sildenafil completely restored spatial learning in the Morris water maze in PCS rats. As shown in Fig. 7a, learning ability of PCS rats treated with sildenafil was identical to control rats. On day 3, PCS rats treated with sildenafil needed 26 ± 4 s to find the platform, which was not different from control rats and was significantly lower (*p* < 0.01) than for PCS rats (Fig. 7b).

**Fig. 8:**
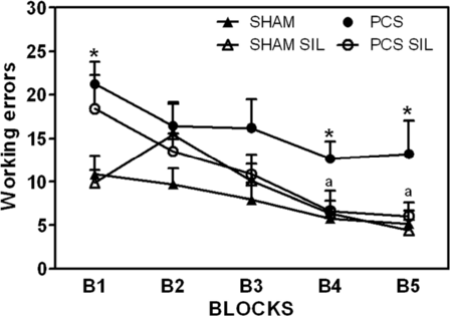
Spatial learning in the radial maze is impaired in PCS rats and is restored by treatment with sildenafil. Spatial learning ability in the radial maze was assessed in sham and PCS rats, treated with vehicle or sildenafil (SIL). Values are the mean ± SEM of 10–14 rats per group. Values significantly different from control rats are indicated by *asterisks* and from PCS rats by *a*. *p <0.05; ^a^ *p* < 0.05

We also assessed spatial learning in the radial maze. As shown in Fig. 8, PCS rats show reduced spatial learning, with more working errors than controls at all blocks. Treatment with sildenafil progressively reduced the working errors in PCS rats and completely restored spatial learning at blocks 4 and 5 (Fig. 8). Working errors in block 4 of tests were 5.8 ± 1.1 in sham rats and were not different (6.3 ± 1.4) in sham rats treated with sildenafil. The number of working errors was increased (*p* < 0.05) in PCS rats to 12.7 ± 1.9 and was completely normalized in PCS rats treated with sildenafil (6.6 ± 2.3). Similar findings were obtained for block 5 of tests (Fig. 8).

### Discussion

The results reported show that rats with MHE due to PCS have neuroinflammation and microglial activation in hippocampus, with increased levels of IL-1β and TNF-α. This is associated with:

1. A strong alteration in membrane expression of GABA_A_, AMPA, and NMDA receptors which would result in altered neurotransmission and
2. Impaired spatial learning in the radial and Morris water mazes.

Treatment with sildenafil normalizes microglial activation and the levels of IL-1β and TNF-α in hippocampus, which is associated with normalization of membrane expression of glutamate and GABA receptors and of spatial learning ability in the radial and Morris water mazes.

**Fig. 9:**
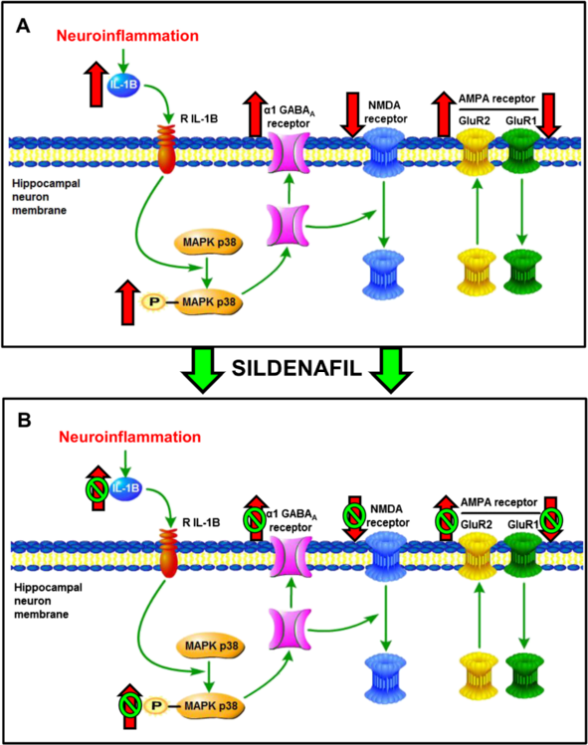
Proposed model for the mechanisms involved in impairment of spatial learning and memory in rats with HE and for their improvement by sildenafil, in hippocampal neuron (CA1) membrane. (**A**) PCS rats show neuroinflammation with increased (red arrow) levels of IL-1β. Over-activation of IL-1 receptor by IL-1β is associated with increased phosphorylation and activity of p38 and increased membrane expression of the alpha 1 subunit of GABA_A_ receptors. The membrane expression of the GluR2 subunit of AMPA receptors is also increased while surface expression of the GluR1 subunit of AMPA receptors and of NR1 and NR2a subunits of NMDA receptors is reduced. (**B**) Treatment with sildenafil reverses (red arrow with green circles) all these changes, returning to values similar to control rats

The alterations found in hippocampus of PCS rats are summarized in Fig. 9. Increased levels of IL-1β lead to over-activation of IL-1 receptor which would enhance phosphorylation and activity of MAP-kinase p38 and lead to increased membrane expression of the alpha 1 subunit of GABA_A_ receptors. The membrane expression of the GluR2 subunit of AMPA receptors is also strongly increased in hippocampus of PCS rats, while those of the GluR1 subunit and of the NMDA receptor subunits, NR1 and NR2a are reduced. These data suggest that neuroinflammation, and particularly increased levels of IL-1β in hippocampus would lead to strong alterations in GABAergic and glutamatergic neurotransmission in hippocampus which would be responsible for the impairment of spatial learning in rats with MHE.

It has already been shown that increased levels of IL-1β in hippocampus impair spatial learning and memory in different situations. Transgenic mice overexpressing human IL-1β in hippocampus show increased IL-1β and neuroinflammation in hippocampus and impaired spatial memory in the Morris water maze [13, 23]. Other situations which increase IL-1β in the hippocampus also result in impaired spatial learning and memory. This occurs for example in post-operative cognitive dysfunction. Surgery of the tibia under general anesthesia in mice caused hippocampal-dependent memory impairment that was associated with increased IL-1 β in the hippocampus. Functional inhibition of IL-1β, both in mice pretreated with IL-1 receptor antagonist and in mice lacking IL-1 receptor, reduced memory dysfunction [24].

Increased levels of IL-1β in hippocampus also mediate the spatial memory deficits in rats injected with the bacillus Calmette-Guérin (BCG) in the hippocampus, which were abrogated by the natural antagonist of IL-1 receptors, IL-1Ra [25]. Increased IL-1β in hippocampus also mediates cognitive impairment following injection of lipopolysaccharide to mimic systemic infection [26] or isoflurane exposure to mimic anesthetic-related cognitive dysfunction in aging [27].

The data in the above reports support that the increased levels of IL-1β found in hippocampus of rats with MHE due to PCS are enough to explain their impairment of spatial learning in the radial and Morris water mazes. Also, the restoration of learning ability of PCS rats by sildenafil would be due to the normalization of IL-1β levels in hippocampus.

A main mechanism by which over-activation of IL-1β receptors in hippocampus led to cognitive impairment is by enhancing GABAergic neurotransmission. Hellstrom et al. [28] showed that in hippocampal slices, lipopolysaccharide increases IL-1β levels and GABAergic neurotransmission. Co-incubation with the IL-1 receptor antagonist, IL-1Ra prevented the LPS-induced enhancement of GABAergic potentials, indicating that IL-1β enhances GABAergic transmission. This enhancement seems to be mainly due to IL-1β-induced increased membrane expression of GABA_A_ receptors. IL-1β enhances GABA_A_ receptor cell-surface expression and GABA-elicited chloride currents by a phosphatidylinositol 3-kinase/Akt pathway in hippocampal neurons [29]. Wang et al. [14] showed that IL-1β, through activation of p38 mitogen-activated protein kinase, leads to enhanced membrane expression of GABA_A_ receptors and of an associated inhibitory current leading to reduced learning ability. Blocking GABA_A_ receptors prevented the impairment of LTP and of learning induced by IL-1β.

We have also found that the membrane expression of GABA_A_ receptor subunit alpha 1 is increased in hippocampus of PCS rats, which could be mediated by the increased activity (phosphorylation) of MAP-kinase p38. This would enhance GABAergic neurotransmission and contribute to the impairment of spatial learning in rats with MHE due to PCS. Treatment of PCS rats with sildenafil completely normalized membrane expression of GABA_A_ receptor, returning to levels similar to those of control rats. This would be a consequence of normalization of IL-1β levels by sildenafil and would contribute to the sildenafil-induced restoration of spatial learning in PCS rats.

As shown in Fig. 5, in addition to the increased membrane expression of the alpha 1 subunit of GABA_A_ receptors, rats with MHE due to PCS also show strong alterations in the membrane expression of NMDA and AMPA receptors, with reduced expression of the NR1 and NR2a subunits of NMDA receptors and of the GluR1 subunit of AMPA receptors and increased expression of the GluR2 subunit of AMPA receptors. These alterations are not due to parallel changes in the amount of the receptors (see Figs. 5, 6), but to altered modulation of membrane expression, likely through changes in phosphorylation.

The reduced membrane expression of GluR1 would be also a consequence of increased IL-1β. Lai et al. [30] showed that exposure to IL-1β reduces phosphorylation in Ser831 and membrane expression of GluR1 in hippocampal neurons, and this was prevented by IL-1Ra, an antagonist of IL-1 receptors. Interestingly, it seems that changes in GluR1 membrane expression would be mediated by NMDA receptors. Blocking these receptors with APV prevented IL-1β-induced downregulation of GluR1 surface expression [30].

As NMDA and AMPA receptors play key roles in LTP and learning, their alterations in PCS rats would also contribute to the impairment of spatial learning in the radial and Morris water maze. The two-fold increase in membrane expression of GluR2 together with the reduction of GluR1 by 44 % leads to a strong increase in the ratio of GluR2/GluR1 subunits of AMPA receptors in the membrane in hippocampus of PCS rats. This would have important consequences on the function of AMPA receptors. Activation of AMPA receptors allows the entry of Na^+^. Receptors lacking GluR2 subunits are also permeable to Ca^2+^ [31]. The presence of a single GluR2 subunit is enough to maximally reduce Ca^2+^ permeability [32]. The strong increase of GluR2 subunits in the membrane of PCS rats will strongly reduce Ca^2+^ entry through AMPA receptors. The reduced amount of NMDA receptors in the membrane will also reduce Ca^2+^ entry in PCS rats through these receptors.

These data suggest that the release of glutamate in the synapsis of PCS rats will lead to much lower increases in Ca^2+^ in the post-synaptic neurons than in control rats due to reduced entry through both NMDA and AMPA receptors. This would lead to strong alterations in the activation of associated signal transduction pathways in PCS rats, which would contribute to impair learning ability.

Normalization of NMDA and AMPA receptors by sildenafil treatment would contribute to restore signal transduction and learning ability.

Sildenafil normalizes IL-1β levels in hippocampus, membrane expression of all receptors and spatial learning in the radial and Morris water mazes. This indicates that it could be a good therapeutic tool to reduce neuroinflammation and improve cognitive function in MHE.

The mechanisms by which sildenafil reduces neuroinflammation would involve cGMP increase and activation of cGMP-dependent protein kinase (PKG). Zhang et al. [15] have shown that transgenic Tg APP/PS1 mice, a model for Alzheimer’s disease, show increased levels of IL-1β in hippocampus and cognitive impairment. Treatment with sildenafil reduces IL-1β to normal levels and restores cognitive function. Both effects are prevented by an inhibitor of PKG, indicating that activation of PKG by cGMP mediates the effects of sildenafil on neuroinflammation and cognitive function. Zhang et al. [15] propose that phosphorylation of CREB by PKG is reduced in Tg mice and its recovery by sildenafil treatment mediates the reduction of IL-1β levels.

The modulation of IL-1β levels by sildenafil may occur at the level of transcription or translation. cGMP modulates directly, through phosphorylation by PKG, the activity of several transcription factors including CREB, ATF-1, TFII-I, and NF-kB [32]. Both positive and negative effects of cGMP have been described for expression of genes for inflammatory proteins (iNOS, COX-2, and TNF-a), leading in some cases to biphasic regulation. Moreover, cGMP may also modulate gene expression post-transcriptionally, by regulating mRNA stability. Elevated levels of cGMP may lead to mRNA destabilization due to downregulation of the mRNA stabilizing factor HuR [33].

IL-1β transcription is mainly modulated by NF-kB [34]. cGMP may enhance activation of NF-kB through a noncanonical pathway involving phosphorylation of NF-kB proteins by PKG [35]. However, cGMP may also reduce activation of NF-kB [36] by a mechanism involving cGMP-mediated activation of heat shock transcription factor (HSF), resulting in elevated HSP70 protein and enhanced binding of HSP70 to IkB, the natural inhibitor of NF-kB translocation to the nucleus and activation [37]. Sildenafil would reduce IL-1β levels by increasing cGMP which may reduce transcriptional activity of NF-kB or IL-1β mRNA stability by mechanisms like those discussed above.

The results reported also support that different types of cognitive alterations in rats with MHE are due to different mechanisms involving different pathways and brain areas and may require different treatments. Impairment of the ability to learn a Y-maze task is mainly due to the reduced function of the glutamate-NO-cGMP pathway in cerebellum, and may be restored by normalizing the function of this pathway [8]. In contrast, impairment of spatial learning and memory is mainly due to the increased levels of IL-1β in hippocampus, leading to altered membrane expression of GABA_A_, AMPA, and NMDA receptors and may be restored by reducing IL-1β levels. This can be achieved using inhibitors of phosphodiesterase 5 such as sildenafil.

These results suggest that inhibitors of phosphodiesterase 5 may be useful to improve cognitive function in cirrhotic patients with MHE. Wang et al. [38] suggest that sildenafil may exacerbate portal hypertension and hyperdynamic circulation in patients with advanced cirrhosis and porto-pulmonary hypertension. However, inhibitors of phosphodiesterase 5 are being used to treat erectile dysfunction in many cirrhotic patients who have not shown secondary effects. Moreover, these inhibitors are also now being used for treatment of pulmonary hypertension and benign hyperplasia of prostate for which new formulations for chronic use have been developed which would be more appropriate to improve cognitive function in cirrhotic patients with MHE without secondary effects.

## Conclusions

The results reported show that rats with MHE due to PCS show neuroinflammation in hippocampus, with increased levels of IL-1β and activity of MAP-kinase p38, which are associated with a strong alteration in the membrane expression of GABA_A_, AMPA, and NMDA receptors and impairment of spatial learning in the radial and Morris water mazes. Treatment with sildenafil normalizes IL-1β levels, phosphorylation of p38 and membrane expression of GABA_A_, AMPA, and NMDA receptors and restores spatial learning ability.

## Abbreviations

MHE: minimal hepatic encephalopathy
PCS: portacaval shunt

## References

[1] Felipo V, Ordoño JF, Urios A, El Mlili N, Giménez-Garzó C, Aguado C (2012). Patients with minimal hepatic encephalopathy show impaired mismatch negativity correlating with reduced performance in attention tests. Hepatology.

[2] Shawcross DL, Davies NA, Williams R, Jalan R (2004). Systemic inflammatory response exacerbates the neuropsychological effects of induced hyperammonemia in cirrhosis. J Hepatol.

[3] Montoliu C, Piedrafita B, Serra MA, del Olmo JA, Urios A, Rodrigo JM (2009). IL-6 and IL-18 in blood may discriminate cirrhotic patients with and without minimal hepatic encephalopathy. J Clin Gastroenterol.

[4] Felipo V, Urios A, Montesinos E, Molina I, El Mlili N, Garcia-Torres ML (2012). Contribution of hyperammonemia and inflammatory factors to cognitive impairment in minimal hepatic encephalopathy. Metab Brain Dis.

[5] Rodrigo R, Cauli O, Gomez-Pinedo U, Agusti A, Hernandez-Rabaza V, Garcia-Verdugo JM (2010). Hyperammonemia induces neuroinflammation that contributes to cognitive impairment in rats with hepatic encephalopathy. Gastroenterology.

[6] Cauli O, Rodrigo R, Llansola M, Montoliu C, Monfort P, Piedrafita B (2009). Glutamatergic and GABAergic neurotransmission and neuronal circuits in hepatic encephalopathy. Metab Brain Dis.

[7] Monfort P, Cauli O, Montoliu C, Rodrigo R, Llansola M, Piedrafita B (2009). Mechanisms of cognitive alterations in hyperammonemia and hepatic encephalopathy. Therapeutical implications. Neurochem Int.

[8] Felipo V (2013). Hepatic encephalopathy: effects of liver failure on brain function. Nat Rev Neurosci.

[9] Butterworth RF, Norenberg MD, Felipo V, Ferenci P, Albrecht J, Blei AT (2009). Experimental models of hepatic encephalopathy: ISHEN guidelines. Liver Int.

[10] Cauli O, Rodrigo R, Piedrafita B, Boix J, Felipo V (2007). Inflammation and hepatic encephalopathy: ibuprofen restores learning ability in rats with porto-caval shunts. Hepatology.

[11] Cauli O, Rodrigo R, Piedrafita B, Llansola M, Mansouri MT, Felipo V (2009). Neuroinflammation contributes to hypokinesia in rats with hepatic encephalopathy. Ibuprofen restores its motor activity. J Neurosci Res.

[12] Monfort P, Erceg S, Piedrafita B, Llansola M, Felipo V (2007). Chronic liver failure in rats impairs glutamatergic synaptic transmission and long-term potentiation in hippocampus and learning ability. Eur J Neurosci.

[13] Moore AH, Wu M, Shaftel SS, Graham KA, O’Banion MK (2009). Sustained expression of interleukin-1beta in mouse hippocampus impairs spatial memory. Neuroscience.

[14] Wang DS, Zurek AA, Lecker I, Yu J, Abramian AM, Avramescu S (2012). Memory deficits induced by inflammation are regulated by α5-subunit-containing GABAA receptors. Cell Reports.

[15] Zhang J, Guo J, Zhao X, Chen Z, Wang G, Liu A (2013). Phosphodiesterase-5 inhibitor sildenafil prevents neuroinflammation, lowers beta-amyloid levels and improves cognitive performance in APP/PS1 transgenic mice. Behav Brain Res.

[16] Raposo C, Nunes AK, Luna RL, Araújo SM, da Cruz-Höfling MA, Peixoto CA (2013). Sildenafil (Viagra) protective effects on neuroinflammation: the role of iNOS/NO system in an inflammatory demyelination model. Mediators Inflamm.

[17] Erceg S, Monfort P, Hernández-Viadel M, Rodrigo R, Montoliu C, Felipo V (2005). Oral administration of sildenafil restores learning ability in rats with hyperammonemia and with portacaval shunt. Hepatology.

[18] Lee SH, Fisher B (1961). Portacaval shunt in the rat. Surgery.

[19] Felipo V, Miñana MD, Azorín I, Grisolía S (1988). Induction of rat brain tubulin following ammonium ingestion. J Neurochem.

[20] Boudreau AC1, Wolf ME (2005). Behavioral sensitization to cocaine is associated with increased AMPA receptor surface expression in the nucleus accumbens. J Neurosci.

[21] Hernandez-Rabaza V, Navarro-Mora G, Velazquez-Sanchez C, Ferragud A, Marin MP, Garcia-Verdugo JM (2010). Neurotoxicity and persistent cognitive deficits induced by combined MDMA and alcohol exposure in adolescent rats. Addict Biol.

[22] Hovens IB, Nyakas C, Schoemaker RG (2014). A novel method for evaluating microglial activation using ionized calcium-binding adaptor protein-1 staining: cell body to cell size ratio. Neuroimmunol Neuroinflamm.

[23] Hein AM, Stasko MR, Matousek SB, Scott-McKean JJ, Maier SF, Olschowka JA (2010). Sustained hippocampal IL-1beta overexpression impairs contextual and spatial memory in transgenic mice. Brain Behav Immun.

[24] Cibelli M, Fidalgo AR, Terrando N, Ma D, Monaco C, Feldmann M (2010). Role of interleukin-1beta in postoperative cognitive dysfunction. Ann Neurol.

[25] Palin K, Bluthé RM, Verrier D, Tridon V, Dantzer R, Lestage J (2004). Interleukin-1beta mediates the memory impairment associated with a delayed type hypersensitivity response to bacillus Calmette-Guérin in the rat hippocampus. Brain Behav Immun.

[26] Terrando N, Rei Fidalgo A, Vizcaychipi M, Cibelli M, Ma D, Monaco C (2010). The impact of IL-1 modulation on the development of lipopolysaccharide-induced cognitive dysfunction. Crit Care.

[27] Li ZQ, Rong XY, Liu YJ, Ni C, Tian XS, Mo N (2013). Activation of the canonical nuclear factor-κB pathway is involved in isoflurane-induced hippocampal interleukin-1β elevation and the resultant cognitive deficits in aged rats. Biochem Biophys Res Commun.

[28] Hellstrom IC, Danik M, Luheshi GN, Williams S (2005). Chronic LPS exposure produces changes in intrinsic membrane properties and a sustained IL-beta-dependent increase in GABAergic inhibition in hippocampal CA1 pyramidal neurons. Hippocampus.

[29] Serantes R, Arnalich F, Figueroa M, Salinas M, Andrés-Mateos E, Codoceo R (2006). Interleukin-1beta enhances GABAA receptor cell-surface expression by a phosphatidylinositol 3-kinase/Akt pathway: relevance to sepsis-associated encephalopathy. J Biol Chem.

[30] Lai AY, Swayze RD, El-Husseini A, Song C (2006). Interleukin-1beta modulates AMPA receptor expression and phosphorylation in hippocampal neurons. J Neuroimmunol.

[31] Liu SJ, Cull-Candy SG (2005). Subunit interaction with PICK and GRIP controls Ca2+ permeability of AMPARs at cerebellar synapses. Nat Neurosci.

[32] Geiger JR, Melcher T, Koh DS, Sakmann B, Seeburg PH, Jonas P (1995). Relative abundance of subunit mRNAs determines gating and Ca^2+^ permeability of AMPA receptors in principal neurons and interneurons in rat CNS. Neuron.

[33] Pilz RB, Broderick KE (2005). Role of cyclic GMP in gene regulation. Front Biosci.

[34] Cogswell JP, Godlevski MM, Wisely GB, Clay WC, Leesnitzer LM, Ways JP (1994). NF-kappa B regulates IL-1 beta transcription through a consensus NF-kappa B binding site and a nonconsensus CRE-like site. J Immunol.

[35] He B, Weber GF (2003). Phosphorylation of NF-kappaB proteins by cyclic GMP-dependent kinase. A noncanonical pathway to NF-kappaB activation. Eur J Biochem.

[36] Kiemer AK, Vollmar AM, Bilzer M, Gerwig T, Gerbes AL (2000). Atrial natriuretic peptide reduces expression of TNF-alpha mRNA during reperfusion of the rat liver upon decreased activation of NF-kappaB and AP-1. J Hepatol.

[37] Kiemer AK, Gerbes AL, Bilzer M, Vollmar AM (2002). The atrial natriuretic peptide and cGMP: novel activators of the heat shock response in rat livers. Hepatology.

[38] Wang YW, Lin HC, Yang YY, Hou MC, Lee SD (2006). Sildenafil decreased pulmonary arterial pressure but may have exacerbated portal hypertension in a patient with cirrhosis and portopulmonary hypertension. J Gastroenterol.

